# Book Reviews

**Published:** 1824-08

**Authors:** 


### CRITICAL ANALYSIS
### OF
### ENGLISH AND FOREIGN LITERATURE
### RELATIVE TO THE VARIOUS BRANCHES OF
### Medical Science.

Quæ laudanda forent, et quæ culpanda, vicissim

Illa, prius, cretâ; mox hæc, carbone, *notamus*.—PERSIUS.

### DIVISION I.

### ENGLISH.

**Art. I.**—*The Principles of Forensic Medicine systematically arranged, and applied to British Practice.* By John Gordon Smith, M.D. Lecturer on Political Medicine. Second Edition, greatly enlarged.—8vo. pp. 569. Underwood, London, 1824.

The first edition of Dr. Smith's work has been for some time before the public; but we do not regret our not having made it the subject of a review when it appeared, as the present edition embraces a wider range of subjects, and contains a considerably larger collection of illustrative facts and observations. Our delay in noticing the work has not arisen either from a mean opinion of Dr. Smith's labours, or an indifference to the importance of medical jurisprudence, as a branch of professional study and cultivation. Our author has the merit of having, in this country, published the first book which deserves the title of a *System* of Legal Medicine, and we hope, in the course of this article, to satisfy our readers that he has discharged his task in a manner creditable to himself, and highly serviceable to the interests of the public and the profession to which he belongs.

The importance of the application of medical knowledge in aid of the righteous administration of the laws, must be evident to every one who contemplates what is passing around him in this world of crime, accidents, and conflicting interests. Cases daily occur in our own Courts, in which the life, the liberties, the rights, and honour of individuals, depend on medical evidence. This alliance between medicine and justice, while it exalts the dignity of the healing art, not unfrequently calls its professors into situations of great responsibility and difficulty. The high importance, to individuals and society, of the interests involved in the exercise of this duty, are, we should think, sufficient inducements to a cultivation of this part of medicine, without our urging, on the medical practitioner, as an additional motive, that his own reputation and fortune may be materially affected by the manner in which he acquits himself on such trying occasions.

It is painful to admit, but it cannot be denied, that the records of forensic medicine contain too many examples where medical testimony, instead of clearing doubts, removing obscurities, and furthering the ends of justice, has involved the case in greater perplexity, and tended either to paralyze the arm of the law, or to direct it to an innocent victim. The attention which has lately begun to be paid in this country to the subject, and the appearance of the present publication, and its successor, the comprehensive work of Dr. Paris, will, we trust, make these mortifying exhibitions among the members of our profession less frequent, by making them familiar with the questions which most frequently call them into courts of justice.

We shall now proceed to lay before our readers the general plan of Dr. Smith's work, and such a view of its contents as our limits will permit. A book nearly extending to six hundred pages, embracing such a variety of subjects, and these treated with as much conciseness as is consistent with perspicuity, does not admit of a copious and regular analysis. We shall, therefore, content ourselves with touching on those divisions which involve the most important questions which a medical man may be required to solve, in his medico-judicial capacity; and this we can, in most instances, do in a general way only, leaving our readers to consult the original for the fuller and more satisfactory details.

The principle of arrangement adopted by Dr. Smith is confessedly not scientific: all classifications hitherto founded on this principle are both imperfect in themselves, and inconvenient in their application to study, reference, and practice;—objects which ought chiefly to be aimed at in forming a classification.

The different questions are arranged under four classes, and these again are divided into sections, chapters, and minor subdivisions.

I. The first class embraces the extinction of life under every variety of circumstance that may render that event the subject of justiciary investigation.

II. Injuries inflicted on the person, not necessarily involving the event of death.

III. Disqualifications, of a physical nature, for the discharge of civil offices, or the exercise of social functions.

IV. Questions of a mixed or miscellaneous kind.

The death of an individual may become the subject of investigation in a court of justice from various causes. That event may take place suddenly, without question of criminal agency, or it may be attended with circumstances calculated to excite suspicions of its being the result of violence inflicted, either by accident, by another person, or by the hand of the deceased himself. These are by far the most important questions relating to medical jurisprudence, and those which most frequently require medical testimony for their elucidation. On this account they occupy the first place in our author's arrangement, and are treated of in the following order:—1. Death in the healthy state. 2. Death where there is question of extrinsic personal agency, or *homicide*. 3. Death where there is question of spontaneous personal agency, or *suicide*. 4. Death of the unborn and newly born, or *prolicide*.

*Death in the healthy state.*—The title of this section is certainly a misnomer, since the majority of the deaths to which it refers result from the fatal progress of diseases, and these of long standing, though their existence may not have been indicated by manifest signs of deviation from the healthy state. The author seems to have been sensible that the title was objectionable, from the explanation which he gives at the outset, that it necessarily implies sudden death, and relates to death occurring without the usual precursory symptoms of that event. *Sudden death*, therefore, would perhaps be more appropriate and correct.

As the reality of a person's death may become matter of forensic inquiry in various ways, the consideration of this important division of the subject is very properly prefaced by a chapter on *apparent* and *real death*.

Marked as is the difference, in general, between the living body and the cold unconscious mass which remains after the vital spark has fled, instances occasionally do happen where life exists, and yet the manifestations of vitality are either suspended for a time, or so obscure as not to be discernible by superficial observers. Cases, also, of real death occur, in which many of the phenomena which usually characterize that state are wanting. Either of these conditions may give rise to investigation and discussion in a court of justice. Many histories are recorded and related of the mere semblance of death having been mistaken for the reality, and fearful consequences are represented as having necessarily ensued; such as, abandoning a patient to his fate when, with care and appropriate remedies, there existed a chance of his recovering; the precipitate application of the scalpel, either for the purpose of anatomical dissection or pathological researches; and, lastly, the interment of a person yet alive. The majority of these histories, however, are to be considered rather as fables than facts, and have had their origin in superstition, credulity, or malice; but, as there is reason to believe that some of them are well founded, (a few of the most authenticated of which are given by the author,) and as the rights to property sometimes depend on the reality of a person's death, and even on the moment at which it may have taken place, medical writers have been at some pains to put us in possession of the means of distinguishing between apparent and real death.

The phenomena usually described as characterizing death are want of voluntary and involuntary motions, insensibility to stimuli, coldness, paleness, fixed state of the eye, contraction of the features, rigidity of the limbs, and relaxation of the sphincter muscles. These different tests are discussed in succession by the author, and the sentiments of various writers as to their validity are given; from which it appears that, if we are to be guided by authorities, none of them can be considered satisfactory proofs; as one or more of them may be wanting in real death, and may exist in subjects who are only apparently dead, and capable of resuscitation. The characteristics most insisted on are the cessation of the vital functions, respiration and circulation, the fixed and collapsed state of the eye, and the rigidity of the limbs. Dr. Paris remarks that respiration, however slow and feeble it may become by disease, must always be perceptible, provided the breast and belly be exposed, when the elevation of the ribs and sternum, and the swelling of the abdomen, can never escape the attentive eye; while our author, on the other hand, quotes Nystens' observation, that the ribs are not raised when respiration is very languid,—that the diaphragm only acts, and that the feebleness of its contractions may be so great as to exhibit no sensible motion in the abdominal parietes.

Louis regards the appearance of the eyes, and the stiffness that occurs after death, as the most evident and decisive proofs of death. Besides the loss of their brilliancy, and the appearance of a glairy membrane, the eyes of the dead, he observes, collapse and grow soft in a few hours,—an alteration which no change in the living body is capable of producing; and he further insists that, as long as the globe of the eye maintains its natural firmness, the person is not dead, whatever other marks may induce us to think so. Desgranges, on the contrary, asserts that the collapse of the eye, and also the glairy membrane, have been observed in persons in a state of asphyxia from various causes, and who have recovered.

The stiffness which belongs to real death must be distinguished from that which may be present in apparent death from cold and convulsions. According to Louis, and others who insist much on the validity of this symptom, in nervous diseases, when the limbs become stiff, the rigidity uniformly precedes the state of apparent death; whereas the stiffness of the corpse does not come on for a longer or shorter period after dissolution. It is also observed that, when the stiffness arises from convulsions, one set of muscles only is contracted, the antagonist muscles being in a state of inaction or relaxation; whereas, in the dead body, muscles producing contrary actions are all in the same condition.

The application of various stimulants, both as tests and means of resuscitation, should never be omitted in doubtful cases, though, as means of discrimination, they are not to be considered decisive. Some have proposed galvanism as a test, regarding insensibility as proof positive of death, and *vice versa;* but, when we recollect that the most violent motions have been excited, by this powerful agent, in subjects who had suffered decapitation, surely little reliance can be placed on it as a test.

The last resource, in all these nvestigations, is the supervention of the putrefactive process, which must put an end to all doubts. The characters already enumerated, however, if properly attended to, will, we should think, enable the practitioner to form a correct opinion; and, where the history of the case, or the appearances presented by the body, leave any doubt on his mind, no appropriate remedies or means of resuscitation are to be omitted before the body is consigned, either to the dissecting knife or the grave.

The states of the living body that are most apt to resemble death, are *asphyxia* from various causes, *syncope*, *apoplexy*, *catalepsy*, *hysteria*, *hypochondriasis*, and indeed exhaustion from any protracted or violent disease. The practitioner who inquires attentively into the history of these cases, and brings a competent knowledge of his profession to their investigation, can be at no loss how to decide and act.

Having discussed the subject of apparent and real death, and the practical application of it to forensic medicine, our author proceeds to the consideration of *sudden death, without suspicion of criminal agency.*

When a person, who was known to have been previously in good or apparently good health, has died suddenly, or is found dead under circumstances of a mysterious or suspicious nature, an examination of the body by a medical practitioner may be required, either for the sake of information and the satisfaction of surviving friends, or at the instance of public justice.

Cases of this description are considered under two heads:—1. Where death occurs from internal causes; and 2. Those in which external, but natural, influences are concerned. To the former head belong several diseases, which may either invade suddenly and prove speedily fatal, or, having existed in an obscure and unsuspected state, for a longer or shorter time, come at once to a fatal termination. Diseases of this nature are generally such as affect one or other of the functions necessary to life,—respiration, circulation, and the nervous energy; such as apoplexy, epilepsy, aneurism of the heart and aorta, or ossification in these organs; rupture of the heart, &c. In some acute diseases, whose first effects are followed by others of a protracted character, sudden and unexpected death not unfrequently occurs,—as phthisis, pleurisy, hepatitis, &c. Inflammation of important organs, either originally situated there, or suddenly transferred to them, as in gouty and rheumatic subjects, and violent emotions of the mind, are also known to occasion sudden death.

The external agents, unconnected with crime, which may occasion death, are lightning,—exposure to noxious gases, to cold and hunger,—the immoderate use of spirituous liquors,—the imprudent swallowing of cold water,—and spontaneous combustion.

After considering each of these different causes in order, and detailing the manner in which they appear to operate on the animal economy in extinguishing life, the author goes on to enumerate the points of duty that devolve on the practitioner, in any sort of investigation that such cases may give rise to.

Inquiries into the manner in which casualties of the foregoing kind have been caused, are conducted by the coroner, an officer appointed by the crown, and a jury summoned by him for the occasion. Before this inquest, all persons who have any knowledge of the matter are summoned, and examined on oath; and it is usual to have recourse to professional testimonies, and, if there be any obscurity about the case, this testimony may be the sole important one. So many interests being involved in the manner, as well as the matter, of his evidence, the researches of the practitioner must be conducted with such care, diligence, and minuteness, as not only to enable him to draw his own conclusions, but also to satisfy others that the grounds of his opinion are correct; and thus leave no room for doubt, cavil, or censure. The following rules to be observed on entering on the duty in question are so judicious, and at the same time so forcibly stated, and so pertinently applied to cases of an intricate and difficult nature, that we will quote the author at some length:

"When, therefore, we are called upon to aid the researches of authority in a case where a person is found lying dead, and no one is forthcoming to give any information on the subject, the following is an outline of what we ought to do.

"It is a rule, under such circumstances, which should universally be observed, that, if the body cannot conveniently remain where found, until an inquest can be assembled, an accurate examination should be made into every appearance connected with it on discovery. It will be of importance to the medical practitioner to know the spot of ground, the situation of the objects, and the posture of the body when found. He must carefully examine the whole surface of the corpse,—not merely to discover wounds or bruises, but also to detect any improper tightness, pressure, or other impediment to free circulation. If there are wounds, they must be carefully traced, and the anatomical relation of the parts in which they are found is to be taken into consideration: if there are bruises, or any marks of violence whatever, the parts beneath must be dissected. These things being premised, whether we have yet discovered any presumptive cause of death or not, we are to proceed to examine the cavities of the body. It is not very material, perhaps, with which we begin; but, in such important cases, we should never be satisfied with the morbid appearances found in one only, however conclusive these may appear. The circumstance of having rested there, and the consequent necessity of admitting the possibility that, by further search, other phenomena, indicating fatal disorder, might have been discovered, may mar the whole process, and subject us to censure. Such neglect has not unfrequently occasioned much perplexity." (Pp. 47, 48.)

The appearances presented on opening the head, which denote death from apoplexy or epilepsy, need not detain us, as these are familiar to every experienced and well-informed practitioner; but the case may be complicated with other circumstances, which demand our earnest attention and discrimination.

"A person, for instance, may be carried off by apoplexy, under circumstances calculated to excite suspicion, either as to the conduct of the deceased himself or that of others, and to throw a mystery over the event, which a judicious examination may entirely remove. For instance, the usual turgidity and discoloration about the countenance may be wanting, while wounds and bruises appear in various parts of the body; and all this, upon careful investigation, may admit of easy and natural explanation. A man may be overtaken with an apoplectic paroxysm in a place where there are hard or sharp objects, upon which he falls, as against furniture in a room, or among stones out of doors: he may thus receive wounds that will appear extensive, and may even lose a considerable quantity of blood. This would be ample cause for popular alarm and clamour, by which the man of science never ought to be swayed. On dissection, the real cause of death will appear, which, along with the extent and nature of the wounds, will show that death has not been produced by external violence. A still more complicated event, however, may occur. It is possible that a person in the apoplectic state may fall, alive, into water, and be taken out dead. In such a case we may expect that the signs of apoplexy will be manifest; and circumstantial considerations must have weight,—such as the nature of the place in which the deceased is found; the appearance of the ground on the margin of the water; the previous state of his mind, health, and general circumstances; as also the degree in which the phenomena of death by submersion exist; of which mention will be made in the proper place.

"Let us suppose another case,—one that, perhaps, may never occur, but which, however extreme, is still possible. A person may fall from a height in a fit of apoplexy, or in any other paroxysm that deprives him of the perception of danger, and of the power of avoiding it. In consequence of this, his skull is fractured, and he is found dead. It can hardly be supposed that the manner in which the fracture has been inflicted, can remain a mystery; it will be seen, from the situation of the body relative to surrounding objects, that this has happened by a fall: but three questions may arise. Has the deceased accidentally come by his death in this way? Or has he sought it of his own accord? Or has he been precipitated by the agency of other persons? Between the question of mere accident and that of suicide, other persons may, perhaps, be as able to decide as ourselves; for dissection may discover nothing but the fractured skull and its consequences. Previous history must here be considered; but it may be that, on opening the cranium, evident marks of apoplexy are found; and further inquiry may lead to the conclusion, that the deceased was seized with a paroxysm of this disorder in a dangerous situation, by which he received the fall in question." (Pp. 49, 50.)

The majority of instantaneous deaths arise from affections of the heart and its large vessels, and the examination of those organs ought to be carried on with great care and nicety; nor must we forget that dangerous and fatal diseases of internal organs, not previously suspected, or overlooked at the moment, may be going on at the time an injury is received; as happened in the case which gave rise to the celebrated Oldham inquest. When these are detected, we have to determine how far the catastrophe was affected or hastened by the injuries inflicted. In examining the alimentary canal, we must bear in mind that we may meet with alterations which may arise from previous disease, the action of deleterious ingesta, or which may have occurred subsequent to the event under investigation.

With regard to death by *lightning*, Dr. Smith observes, that morbid anatomy, without any knowledge or suspicion as to the agency of electricity, will serve us little in the investigation. Few dissections of those who have perished in this manner have been made; and, except the scorching on the surface, and the discoloration said to be particularly observable in the direction of the spine, all the appearances described have been found in the bodies of those who have died from other causes. Death by lightning has been said to take place *qua* suffocation. Mr. Brodie, from opening the bodies of animals killed by electricity, concludes that death takes place in the same manner as from a severe injury of the head.

The case of death from *noxious gases* may be involved in difficulties of various kinds, which are ably stated by the author.

"We may readily suppose that carbonic acid gas may have been inhaled to a fatal extent; yet, when the body is found, the circumstances as to noxious influence may be altogether changed. For instance, a person goes into a cellar, and has occasion to stoop, in order to accomplish the purpose for which he entered. The accumulated gas, which is specifically heavier than the air of the atmosphere, is at the time high enough to deprive him of sensibility, and, falling still lower among it, he perishes. Some time elapses before he is discovered, while the gas, having found an outlet at the open door, has escaped; and a burning object will then be supported at the very lowest part of the place. In such circumstances, perhaps, there will be evidence enough of what has occurred, from the state in which objects are found. The process by which the evolution of so much gas had been produced, may be found going on: the previous closeness of the door may be attended to,—the length of time during which it had been unopened may be recollected, and the whole event be satisfactorily explained. But we may find persons killed by this gas, under circumstances of a more doubtful nature. The eyes of such as die in this manner are generally found wide open, and, as some say, protruded from the sockets. The tongue is commonly thrust out, and that at one side of the mouth; the jaw at the same time clenched, and the face livid. We may not find marks of violence, unless where there has been a fall from a height; and then the nature of the external injury will explain itself, in connexion with the other circumstances.

"On opening the body, we shall find the proximate phenomena, if I may so express myself, of suffocation. What is meant by these will be discussed hereafter. I shall merely remark now, that there will be a congestion of blood in the right side of the heart, and in the veins leading into those cavities. Where this exists, and no other cause of suffocation is demonstrable, such as drowning, strangling, or morbid impediment, we have proof enough, when added to the history of the event, to decide in what way a person in such circumstances has been carried off." (P. 54—56.)

In mortalities from *hunger*, we shall not only find emaciation and emptiness in the stomach and intestines, but very scanty remains of blood in the vessels; which, with the previous history of the circumstances of the deceased, together with the absence of other morbid appearances, will point out the cause of death.

Death from exposure to *cold*, in inclement seasons, frequently occurs in this country, and becomes a subject of investigation before the coroner; but our author is not able to produce any thing satisfactory as to the appearances presented in such cases. We shall generally find that the subjects of such casualties were in circumstances of want and misery, or may have themselves contributed to their fatal end by excessive drinking; or, at least, such indulgence as has rendered them unable to resist the fatal influence of the exposure.

Death from a sudden draught of *cold water*, when the body is in a state of great excitement from violent exertion, may take place either instantaneously, or in consequence of disease induced. The former consequence is ascribed to a powerful sedative impression made on the nervous system, through the medium of the stomach; and, from the rapidity of the fatal action, it is supposed that no corresponding lesion can be expected to be found in the dead body. In these cases, we must depend on the evidence derived from the history of the circumstances, and the absence of marks indicating any other cause of death.

In cases where death is presumed to take place from *intoxication*, we look for apoplectic symptoms; but sudden death may take place from large potations, without leaving any perceivable trace of internal derangement. In all these cases, we may expect to meet with the presence of the liquor in the stomach and intestines.

The last subject noticed under this division of the work, is the *spontaneous combustion of the human body.* The fact itself has, in this country at least, been generally disputed; but our author thinks the cases recorded of such occurrences so respectably attested, that it can no longer be doubted. Instances are mentioned where persons were accused and condemned on a charge of murder, in consequence of bodies having been found consumed in this manner; and, as the occurrence might be urged as a defence in cases where attempts have been made to burn a body after murder, in order to destroy all evidence of crime, and thus the question might become the subject of judicial discussion, our author details the phenomena alleged to attend it, and distinguish it from ordinary burning. He also gives a brief notice of the best authenticated cases on record, and refers to others.

The questions arising out of cases in which the extinction of life in one person is effected by the agency of another, are by far the most frequent causes of our appearance in a court of justice, and are those in which the most serious consequences depend on our mode of exercising the duties that are required of us in such situations. This section embraces the important subjects of *poisoning*, *suffocation*, and *wounds and bruises*, or injuries analogous to them.

Dr. Percival remarks that, on such occasions, the medical practitioner should be qualified to give his testimony consonant to legal, as well as medical, knowledge; that he must not only be acquainted with the signs of natural death, as well as those which occur when it is produced by accident or violence; but that *he should not be a stranger to the several distinctions of homicide established in our courts of judicature.* Now the latter qualification here required of the medical witness, we do not consider at all essential, notwithstanding the high authority quoted, and the sanction which our author seems to give to the opinion, by detailing, both in the text and more at large in the Appendix, the several distinctions in question. The duty of the practitioner is simply to ascertain the manner and cause of death; and we do not see how the investigation of these facts can be influenced by a knowledge of the degree of culpability attached, by the law, to the act which occasioned it.

The only qualifications necessary to the practitioner for the proper performance of such a duty, are a thorough knowledge of his profession, and the exercise of a sound judgment. On entering upon it, he ought, as much as possible, to divest himself of any impressions he may have received from rumour or popular clamour; bearing in mind that the fatal event which he is about to investigate may turn out to be the result of accident or natural causes, and not the consequence of criminal agency. Should his researches lead him to conclude that life has actually been destroyed by unfair means, he may encounter some of the following questions:

"Are there no fatal diseases that leave appearances similar to those found in the body on the present occasion? Could the person have inflicted this injury on himself? Has not a fatal result taken place in this instance, from a cause that in another person would have been of little or no consequence? Was every thing done for the recovery of the deceased? Or, might he not have recovered, had proper treatment been pursued? And in certain cases, as hanging and drowning, it has been matter of inquiry, whether the person was killed in the manner alleged, or first deprived of life, and then placed in that situation in order to baffle suspicion? All these, and other questions of equal importance, have been repeatedly put to professional witnesses." (P. 63.)

The subject of *poisoning* receives from our author the ample consideration which its importance in medico-legal investigation necessarily claims. Articles which possess this fatal quality may be administered with a criminal intention on the part of others, or resorted to by wretched individuals to take away their own lives. They are also frequently mistaken for substances of an innocent or useful nature, employed in food or medicine; and sometimes persons are exposed to their deleterious influence from these articles being employed in the arts, or entering into the fabrication of utensils.

Poisons differ from each other in their physical and chemical characters, as well as in the effects which they exert on the living organized system; and these differences constitute the basis of the two most common arrangements, or classifications, adopted by systematic writers,—that which is founded on the basis of natural history, and that which classes them according to their effects. Dr. Smith avails himself of both these principles of arrangement, discussing the poisons belonging to the mineral, vegetable, and animal kingdoms, in the order of their active relations, as *corrosive* or *escharotic*, *astringent*, *acrid*, *narcotic* or *stupifying*, *narcotico-acrid*, and *septic* or *putrifying*.

Before entering on the particular examination of the individual poisons, the author gives a brief sketch of the symptoms which characterize the action excited by each of the above classes on the living system, and the morbid lesions resulting from them, and discernable on dissection after death; concluding with some useful rules, which are generally applicable, for the guidance of the practitioner, when called upon in emergencies of this kind.

"In the investigations which it may be our duty to perform, we may have to do either with the living or the dead body. In those cases to which we may be called during the life of a person who has taken poison, the primary object of the practitioner must, of course, be to save his patient, and remove suffering; and, though successful in both of these indications, it may not be the less necessary to institute a judicial inquiry into the facts connected with the case. As it must, therefore, be of the utmost importance to know what particular poison was administered, it is incumbent on the practitioner to be careful, in his endeavours to save life, not to destroy the means of verifying or disproving an imputation of guilt.

"One rule to be invariably observed when we are called to a case of poisoning, is to ascertain, if possible, what the individual last swallowed, to get possession of the utensil in which it has been contained, and of the remains, if there be any. If the person has vomited, it is of equal importance to secure the rejected contents of the stomach.

"If we are called while the sufferer is yet alive, we must also observe the symptoms; and a corroborative proof, as to the particular deleterious article administered, is to be obtained from the successful application of an established antidote.

"Having obtained a portion of the substance in which the poison has been mingled, or of the matter vomited, we may submit a part to such tests as can be most readily procured; the immediate result of which may assist us in our decision as to the proper practice for recovery. In order to speak to the purpose, however, in a court of justice, the mass should be reserved for deliberate experiment, when we have the means of insuring accuracy.

"It has been recommended to give part of the suspected substance to an animal, and much stress has been laid, and a good deal of discussion employed, upon this point. I shall content myself with saying, that the loss of time, and probably of material for more accurate investigation, would generally be great and evident objections; the rather, as we cannot, by this experiment, propose to identify the individual poison.

"In every case of suspected poisoning, where the history is unsatisfactory, it will be of considerable importance to inquire into the patient's habits of life, peculiarities of constitution, and liability to complaints, as well as the nature of these; whether he is known to have been formerly affected in a similar manner; whether particular articles of food, generally considered wholesome, used to disagree with him; of what his last meal consisted; and whether any mistake, or deleterious interference, could have occurred in the culinary department.

"These injunctions are palpably necessary where the person is yet alive, and the great object is recovery; but they should also be attended to where death has taken place. The only object we have then in view is the detection of the poison; and, being masters of time, and having it in our power to carry investigation much further, and to pursue it more deliberately than during life, we must be careful to throw no obstacles in the way of a full elucidation of the question, by hurry or impropriety in our mode of proceeding.

"We are to seek the cause of death, also by negative as well as positive proofs. By these I mean the absence of all other signs of injury or derangement, whether from violence or disease, that might be urged as the cause of death. The presence of the fatal substance, and the known or presumed effects of such substance on the system, form what may be termed positive proofs.

"In searching, post mortem, for the presence and traces of poisonous ingesta in the internal parts of the body, the following rule of procedure will be applicable in all cases. The whole canal for alimentary functions should be examined, from that part of it which is appropriated to deglutition to the ejecting extremity; laying open the fauces and œsophagus, and examining the large intestines as well as the small.

"We are not to be deterred from this examination, merely because there may have been an inconvenient lapse of time between the death of the individual and the period of investigation. It was formerly believed that, after a few days, any researches in the body would be useless, and even dangerous. With regard to the danger, it may be observed, that much is placed to this account that belongs merely to disgust; and, as to our being baffled in the search, if it be rightly conducted, the detection of mineral poisons, at least, cannot be affected by the approach of putrefaction." (P. 76—79.)

The mineral kingdom supplies by far the greatest proportion of the poisonous articles which are either intentionally administered or accidentally taken; and under this division are included *metals* in the states of oxides and salts, *acids*, *alkalies*, *earths*, and the combinations among them usually denominated *salts*, or *neutral salts*. It comprehends most of the corrosive poisons, all the astringent, and some of the acrid.

The author begins with the metals which, in certain states of combination, become deleterious to human life; and *arsenic* naturally heads the list, followed by *mercury*, *copper*, *silver*, *antimony*, *zinc*, and *lead*.

Each of these is treated of, first, as an object of natural and chemical history: its specific action on the constitution, whether administered as medicine or poison, is then considered,—its established antidotes,—the morbid appearances observed on dissection, when death is the result,—and the most certain and approved tests by which it is recognized, with other circumstances, of a collateral nature, which may aid us in our investigation; lastly, the practical application of the knowledge we possess on these points is laid down.

The consideration of the individual poisons under these several aspects is necessarily so extensive, that we cannot, in the short space allowed us, attempt to follow the author, without swelling this article to an undue size, or presenting our readers with only a meagre and unsatisfactory detail of a portion of the work, replete with information of the first importance to the medical practitioner.

[To be continued.]

---

### DIVISION II.

### FOREIGN.

ART. II.—*Consultations et Observations de Medicine, de feu* CH. L. DUMAS, &c. &c. Publiée par le Dr. ROUZET, Membre adjoint de l'Academie Royale de Medecine; Medecin de la Mounoir Royale des Medailles, &c. &c.—pp. 498. Paris: chez Gabon et Compagnie. 1824.

*Consultations and Cases in Medicine, by the late* CH. L. DUMAS, &c.

PERHAPS a more useful bequest could not be left by an eminent medical practitioner to the profession in general, than a work which should contain a candid account of the errors into which he had fallen during the course of his practice, and an undisguised history of those cases in which he had been unsuccessful, either from the nature of the case itself, or from having formed an erroneous diagnostic. Such a publication could only be a posthumous one, since it is not to be expected that any man, in his life-time, continuing to practise his profession, the basis of his reputation being founded upon public opinion, could venture to record his mistakes.

The work before us is a posthumous one, but it is sadly deficient in two points. In the first place, the consultations are not followed, excepting in two or three instances, by any subsequent account of the progress of the cure; and the cases are almost all fortunate in their termination, bearing, therefore, the marks of having been selected from a large number; and that very selection being, as we have hinted above, prejudicial to the progress of science. Although by no means inclined to think well of the state of medical practice in France, we are induced to give some account of this volume, because the diseases of which it treats are principally chronic, a class of maladies to which M. DUMAS had paid particular attention, and in which the minute care bestowed upon diet and regimen, as well as the employment of those obsolete and old-fashioned remedies still in vogue amongst them, are occasionally crowned with a success which would not at first sight be expected, or believed, by those accustomed to the more energetic prescriptions of English practitioners; and which, in the treatment of the acute forms of disease, renders us so much superior to our continental neighbours.

Dr. ROUZET, the editor of this volume, informs us, in a short preface, that these cases were destined to form part of a clinical work, which M. Dumas intended to publish, as an illustration of his general doctrine of chronic diseases. He also meant to have accompanied these cases with remarks on the principles which they were intended to establish. With regard to the Consultations, which indeed constitute two-thirds of the work, we are informed that they are extracted from two select Books of Consultations, from whence he proposed to have drawn the materials for his clinical work, the greater part of these consultations having been crowned with complete success. We can only repeat, that we should not regret giving up half the fortunate cases for the sake of those which terminated fatally.

In making our selection from this volume, we shall pass by those cases and consultations in which we conceive our author to have erred in principle, and consequently in practice; but dwell more at large upon those points which, however minute, we conceive are occasionally too much disregarded by practitioners on this side the Channel.

The first consultation which we shall translate is the seventh in the volume, and is entitled "Weakness of the Brain, and of the Organs of Generation." The subject of this Memoir had been addicted to the practice of masturbation from the age of fifteen, and which, together with hard study and inquietude of mind, had produced a greater effect on a temperament disposed to melancholy. Impaired digestion, weakness at the chest, irritability, and rheumatic pains, were the most obvious symptoms. At the age of twenty-two, the patient had the venereal disease, to which the pains in the limbs are attributed, but generally he was improved in health, although living in the midst of agitation and fatigue, both of body and mind. The complaints for which he applied for advice are—first, a strong propensity to sleep, principally felt after his meals; and which our author decides to be owing to the state of the stomach and intestines, rather than to that of the brain itself: the second complaint refers to the parts of generation, which are so enfeebled as to be useless. M. Dumas considers this to be a branch of the same nervous weakness which appears to have affected the brain and the viscera of the abdomen, and, as depending upon the same principle, requiring to be combated by the same means. Besides this, he is of opinion that some particular vice of the semen itself renders its ejection premature; and this alteration he conceives to be derived from the vitiated condition of the digestive organs, by which its constituent principles are elaborated. To remedy these defects, the following plan is pointed out:—

1. The patient's nourishment to be mild, full of gelatinous and nutritive matter, and especially that which contains the greatest quantity of nourishment in the least space; and, after an enumeration of various dietetic articles, hartshorn-jelly is recommended, both as food and medicine. He is recommended water and a mixture of old and dry wines as drink, but to be taken cold, even of the temperature of ice; and he is to abstain from fermented and spirituous liquors. He must go to bed, and rise early. With respect to exercise, he is to be moderate; that on foot is preferred to horse-exercise. He must avoid much labour of the mind, and every thing that stimulates the imagination. Without exciting useless desire, he may, however, be permitted to make an essay of his powers occasionally; and add to the regimen above mentioned the following curative means:—1st. A cold bath every morning, followed by a sufficient quantity of exercise to produce a gentle moisture of the skin. In the evening, when the tendency to sleep comes on, he will cause cold water or ice to be applied to the head; the neck and spinal column is to be rubbed with brandy, mixed with some volatile alkali; and the limbs are to be rubbed, morning and evening, either with a flannel or brush,—particularly the abdomen, loins, and the neighbourhood of the genital organs; to which latter he will apply ice every day, and wash them with a solution of white vitriol and alum in water, with some tincture of cantharides added to it, (the proportion mentioned is only fifteen or twenty drops,) to the quantity necessary.—2d. At each meal, the patient is to swallow, with the first spoonful of soup, one grain of ipecacuanha in powder; and, after the meal, a spoonful of the elixir of Hoffman.—3d. He will vary the use of stomachics and tonics,—such as cascarilla, chamomile, &c.; together with mineral waters containing steel,—such as those of Lamalon and the neighbourhood of Uzes.—4th. Every morning and evening, the patient will take the quantity of a nut of a confection, the principal ingredients of which are bark, aloes, saffron of Mars, and powder of arnica; and this prescription may also be made into tablettes, so that he may take some of them in the course of the day.—Finally. The utility of all these remedies may be further promoted by the application of a plaster to the perineum, composed of turpentine, white vitriol, and terra sigillata. Asses' milk is hinted at as likely also to be serviceable in the progress of the cure.

Such is an abridged outline of the treatment proposed in this case; and we have the authority of the editor for the successful result of its application, since he informs us that he found notes to that effect among the papers of M. Dumas.

We shall offer but few remarks upon this case: indeed, we must leave that task to our readers, as the space we can devote to the work will not permit of our doing so; and proceed to notice the next Consultation, which appears to us to afford some points of interest. We may, however, observe, that the principles upon which M. Dumas proceeded in the treatment of the above disease were radically good, though some of his remedies were puerile, and others of very doubtful efficacy. With regard to the effects produced upon the constitution by the practice of masturbation, two very opposite opinions have been maintained by writers of nearly the same period. Tissot drew a most frightful and overcharged picture of the ill consequences attending it; whilst John Hunter treated it as a practice, physically speaking, attended with no particularly alarming results. The truth certainly lies between these extremes: if the habit is broken early in life, there can be no doubt that the constitution will finally triumph over the symptoms of debility which it always induces; and that, in the lapse of time, and with proper management, the sexual organs will recover their tone and efficiency; but it is no less true that, in strumous habits, the foundation of many a fatal disease is laid by a perseverance in this degrading custom, and that consumption, especially, is too often the result.

The 16th Consultation is a case of *laryngeal phthisis*, the consequence of repeated catarrhal affections, probably aggravated by the closure of a fistulous ulcer, which the patient (a young lady) had been affected with in the chest; and this M. Dumas considers the more likely, as the constitution is lymphatic, approaching to scrofulous. The symptoms mentioned are—alteration of the voice, a fixed pain in the throat, a pain in deglutition, an impression of heat felt at the bottom of the larynx, and an expectoration of a suspicious matter; and these combined lead the author to conclude that the larynx and trachea are alone affected, and that the lungs are still sound. But the diminution of strength, the emaciation, and continued fever, announce the progress of the disease, which appears to have reached its second stage; the difficulty of swallowing tends to the belief that the mucous membrane of the trachea and larynx is diseased, and that there are points of ulceration established in this part.

The three indications to be observed in the cure of this disease are—to calm the irritation of the throat, to turn aside the fluxion, and to prevent the formation of lymphatic congestion and ulceration. To fulfil these intentions, the patient is directed to use, for a common drink, a strong decoction of coltsfoot mixed with gum arabic; and to take every morning a broth, which bears a close resemblance to mock asses' milk, and which is afterwards to be substituted for it. Before this treatment is commenced, the patient is to be purged with an infusion of rhubarb, manna, and cream of tartar; and this to be repeated every fortnight:

this is meant as a revulsive remedy. She is also to use injections composed of decoction of chamomile mixed with a little soap, and made still more irritating by the addition of some sal ammoniac; for the same purpose, a pediluvium is also to be employed, some salt and mustard being added to the water. Pastilles of the leaves of aconite in powder, half a grain in each, are to be swallowed every three hours. To calm the cough, and to allay the irritation of the throat, several anodyne remedies are recommended to be taken at night,—either poppy infusion, the juice of lettuces, or opium. Fumigations of amber and mastic are also to be used to the throat; as well as the vapour of pectoral and detersive plants, and more especially the inspiration of sulphuric ether, in which some leaves of cicuta are digested. Three or four leeches are directed to be applied to the painful part of the throat; to be followed by a blister, which is only to remain long enough to irritate the skin. The situation of the blister is to be frequently changed from the neck to between the shoulders, then to the chest, and finally to the inner part of the thighs. If any appearance of pain, swelling, or inflammation about the parotid glands, should come on, M. Dumas recommends suppuration to be encouraged; or, should there be a wound in any part of the body, he advises it to be carefully kept open; and the lymphatic temperament of the patient makes it necessary to mingle the above treatment with the exhibition of tartarised salts, antimonials, balsamics, and tonics,—even the cascarilla and cinchona. The diet is to be that which comprises much nourishment in a small space.

This case may give rise to many reflections; and there may be good reason to believe that some of the remedies recommended by our author are now too much neglected. The plan of inhaling the vapours of medicinal substances once was in great vogue, and, if we may believe our countryman, BENNETT, the author of a paper in the "Memoirs of the French Academy of Surgery," and others, it was occasionally productive of much good. Lately, Sir A. CRICHTON has revived the practice in favour of tar; and, though his praises of its good effects may be considered as overcharged, yet, in certain instances, no doubt can be entertained of its having been followed by successful results. The cases in which this practice appears available, are those in which the larynx and trachea alone are affected: nor does it appear probable that the serious affections of the lungs themselves can be influenced by such means. The paper in the fifth volume of the "Memoirs of the French Academy of Surgery," deserves to be consulted with reference to this practice.

We now pass over several Consultations, and stop at the 40th, a *case of epilepsy from worms.* M. Dumas forms this diagnosis from the circumstance of several individuals of the family having been thus affected. The first appearance of epilepsy took place in consequence of a fright, and was repeated at very short intervals, but without any regularity. The fits were long and violent, sometimes lasting twenty-four hours. Each attack appeared to commence at the stomach, and mount to the head; and, at its termination, the patient passed by stools both ascarides and lumbrici, and vomited some of these latter worms, together with a quantity of glairy matter. This patient had been much relieved by vermifuge remedies; but, upon the approach of puberty, the disease resumed fresh force, an alarm reproduced it, and the symptoms became more severe than even at first. By the advice of the physicians at Paris, a decoction of aloes, with nitre, was administered; which simple remedy, by discharging a quantity of ascarides, diminished both the number and violence of the attacks. From this exposé, our author infers that two principles tend to keep up the disease; the first being an extreme activity of the nervous system, and the other the influence of *diathese vermineuse;* this last principle proving the existence of a singularly strong tendency to the degeneration of the humours which nourish and reproduce these worms. The method of treatment, therefore, resolves itself into these two points:—

1st. To moderate the action of the sensitive and moving powers, by procuring, by well-directed excitation, a free development of those powers. 2d. To destroy the superabundance of mucous fluid, and to prevent the generation of the worms which is the consequence; and this last indication is the most important. In addition to the means usually employed against nervous debility,—such as warm baths, dry rubbing, &c.—the skin ought to be *animated* in different parts by the application of blisters, which are not to be left on long enough to produce a wound; and frictions with opium, camphor, and amber, dissolved in spirit of wine, are to be employed upon the thighs, legs, back, and along the spinal column. The principal treatment will consist in causing the patient to vomit three or four times on alternate days, by means of a dose of tartar emetic and ipecacuanha; then the patient is to be purged twice in the month, with a composition of cinnabar, guaiacum, jalap, rhubarb, and diaphoretic antimony, mixed with chicory water. In the intervals, he is directed to make use of pills composed of mercury, fern powder, camphor, valerian, assafœtida, and aloes. The coming on of the epileptic paroxysm is to be narrowly watched; and, if it is foreseen, vomiting is to be produced by glysters of salt and water, and by drinking warm water; and the glysters may continue to be employed in the intervals of the attacks. If, after the worms are entirely got rid of, the epileptic paroxysms continue, they must be combatted by the use of direct and appropriate remedies, principally antispasmodics. If, says our author, by the effect of these remedies, or by some salutary and unlooked-for change, a feverish attack should come on, it would be advisable to favour and maintain it long enough to resolve the epileptic disposition, since fever has occasionally been found to cure this complaint. The same observation extends to collections of matter, cutaneous eruptions, &c. The regimen is directed to be tonic and fortifying. The patient must avoid all sudden changes of the weather; must avoid cold and damp situations, take a moderate quantity of exercise daily, and avoid all fat, unctuous, mucilaginous food.

Having now given a few specimens of our author's mode of disposing of his Consultations, we shall turn to the latter part of the volume, and extract one or two Cases; and which we do with the more pleasure, as we are enabled to give the results of the measures proposed, which, with one or two solitary exceptions, the Consultations do not afford.

The first case which we shall extract is that of a lady, thirty-four years of age, who was affected with a nervous head-ache, constantly aggravated at each period of menstruation.

The patient had lost both flesh and strength, had become very irritable; the abdomen was slightly tumefied, and this enlargement increased at the menstrual period; a sense of general uneasiness; slow and imperfect digestion; a hard, contracted pulse; respiration impeded; and palpitations of the heart, when the pain was violent, were the most marked symptoms. A sense of weight in the loins, and a creeping sensation in the thighs, always accompany the periods of menstruation, which are often interrupted and imperfect.

The treatment of this case consisted in obviating the irritability and moderating the faulty action of the uterus: for this purpose, a bleeding of four ounces was directed immediately after the cessation of the menses; the next day, an opening medicine; and, on the third, baths, and a bolus of four grains of camphor, six of castor, and one of opium. In addition to these baths, a pediluvium is recommended every evening; and an injection of a decoction of valerian and chamomile, with a few drops of laudanum, every second or third day. These remedies were continued for two months; frictions upon the internal parts of the thigh being performed six days prior to the expected return of the menses; then the bathing of the legs is to be more assiduously employed, and leeches are to be twice applied to the upper part of the thighs. Four glasses of an infusion of the flowers of mugwort, ten drops of tincture of aloes, and as much of the aperient tincture of iron, are given every day. After the lapse of two months, all the symptoms had given way in a great degree. The above remedies were continued; and to these were added pills made of valerian, aloes, henbane, and opium, gradually augmenting the dose, under the use of which the patient soon got entirely well.

The sixth case is an example of *epilepsy*, cured by an attack of fever, attended with a copious discharge of stools.

C. B., twenty-eight years of age, of a pituito-sanguine temperament, born of healthy parents, and possessing all the external marks of a healthy constitution, had enjoyed excellent health until his twentieth year, when he suffered from a violent peripneumony, accompanied with delirium and subsultus tendinum. The year following he had hospital-fever, which terminated favourably in eighteen days. After having joined the army three or four years, he saw several of his companions suddenly destroyed by the explosion of a mine. Immediately he experienced an universal trembling, and two hours afterwards had a violent attack of epilepsy, and which were renewed every day, at the hour when the explosion took place. From that period until his entering the hospital, on the 4th March, 1805, the disease presented no change: the attacks are sometimes more severe and of longer duration than at others, sometimes lasting five minutes, occasionally extending to a quarter of an hour; they consist principally of convulsive agitation and shaking of the upper and lower extremities, accompanied with contractions of the muscles of the face, loss of recollection, &c. In the intervals the patient is tolerably well: nevertheless, he complains commonly of a violent pain in the head, and a lively pain in the epigastric region. His countenance was melancholy; he sighed involuntarily; exhibited great lassitude; his sense of hearing was remarkably acute, the least noise producing a general tremor; the heat of the skin natural; pulse strong and full; respiration free, but sometimes interrupted, as if the blood found a difficulty in passing through the lungs. The stomach is often deranged, with little appetite and slow digestion, and costive bowels. The sexual organs do not feel the influence of this disease in any way.

Two methods, continues M. Dumas, present themselves to restore the harmony of the system: the first consists in procuring a calm of the nervous system, by moderating the influence which the affection of the head appears to exercise over the whole of the economy, and especially upon the epigastric organs; and revulsives and calming medicines will produce this effect. The second plan is to impress upon the system some violent shock, which, by a sudden revolution, may change the morbid relation between the sensibility and contractility of the system, and re-establish their harmony. Emetics and sudorifics are recommended for this purpose. On the 6th March, the first method was acted upon. Baths of the feet with mustard, bleeding in the foot, and frictions over the whole body, were resorted to, and continued until the 10th, excepting the bleeding. During these four days, an attack of epilepsy came on daily at noon; that of the 9th lasted a quarter of an hour, and left a convulsive motion of the lower jaw, such as is seen in the cold stage of fever. On the 11th, the foot-baths, injections, frictions with tincture of cantharides, fumigations, a grain of opium four times in the day: the last remedy intended to allay the pain in the head, which is supposed to proceed from nervous excitation. On the 13th, an attack of epilepsy, as violent as usual, supervened; the pulse high, face red, &c. Four leeches were applied to the inner part of each thigh; the opium is continued, and the baths with mustard. On the 14th, the attack was more moderate; the same remedies were repeated, excepting the leeches. On the 16th, they were re-applied, and the same treatment continued: there was no attack of epilepsy. From the 17th to the 25th, the baths are continued, and the patient is purged twice with rhubarb and jalap. The attacks came on each day, excepting the 21st and 22d. On the 26th, a different treatment was adopted: three grains of tartar emetic produced a considerable vomiting of glairy matter; in the evening, perspiration was produced by a sudorific potion. Every two days, the emetic and sudorific was repeated. On the 16th of April, another dose of emetic was given: bilious vomiting ensued, and abundant sweating, without any sudorific medicine. On the 13th, an accession of fever took place, which lasted twenty-four hours, and terminated by copious black-coloured evacuations.

After this, the patient continued several months in the hospital, but had no return of his disease.

We have only one remark to make on this case, which is this —that all the former part of this treatment was not only lost time, but, from the state of the pulse, violence of the pain in the head, and other symptoms, much danger was probably incurred; and we doubt not that a moderate bleeding in the first instance, with a free use of purgatives and of emetic tartar, would very speedily have cut off the malady.

We shall conclude our extracts by a detail of the 13th Case, *palsy of the upper extremities.*

N—, aged seventeen years, of a robust constitution and sanguine temperament, accustomed to hard labour, and hitherto healthy, was suddenly struck with palsy of the upper limbs: he was enjoying perfect health at the moment of the attack, and presented all the exterior signs of strength and vascularity. The loss of motion was more complete on the right than the left side; the integuments preserved their sensibility; the pulse more developed on the side which had entirely lost its motion than on the other side; the intellectual faculties as usual; and respiration much impeded. These symptoms our author refers to two causes: 1st, to sanguineous congestion; the 2d, which is in fact only the consequence, refers to the loss of the power of muscular contraction in the members affected; and these two causes lead him to adopt two indications of cure.

On the 11th March, 1808, five days after the patient's admission into the hospital, the first order of remedies, against the plethoric affection, was commenced by a bleeding from the foot, to the amount of eight ounces, and baths to the legs, in which salt and vinegar are dissolved; internally, whey and nitre is given. The next day, six leeches are applied to the inner part of each thigh; the same remedies being continued. On the 14th, eight leeches are applied to the anus; and on the 16th, twelve ounces of blood taken from the foot. From thence to the 25th, the leeches and bleeding are again repeated; and then the patient's countenance loses its high colour, and he felt less weight and a sense of amendment; the leeches and bleedings are therefore suspended. From the 25th March to the 20th April, frictions along the paralysed members and vertebral column with tincture of cantharides, are recommended. Sudorifics and exciting medicines are also administered. The powers of contraction are gradually resumed; the strength returns; and the patient, after taking some slight doses of an infusion of arnica, leaves the hospital perfectly cured, after two months of treatment.

We have now given our readers a fair specimen of M. Dumas' practice: the nature of the work, consisting entirely of detached cases and consultations, prevents us from doing more than this. There are no general principles upon which we can comment; and, in conclusion, we can only observe, that, however different the practice appears to be to that which we are accustomed to witness, and however puerile and trifling some of the remedial agents which our author depends upon may appear, in many respects the work will repay the trouble of a careful perusal, and afford some hints which we, in our eagerness to act from great and general principles, are too apt to overlook, in the management of chronic and complicated cases.

