# Errata in Our Last Number

**Published:** 1824-09

**Authors:** 


					1 age 142, line 18, for head read heart.

				

